# Supplementary material for: Special Attention to Physical Activity in Breast Cancer Patients during the First Wave of COVID-19 Pandemic in Italy: The DianaWeb Cohort
Source: J Pers Med. 2021 May 6;11(5):381. doi: 10.3390/jpm11050381 (PMC8148515; doi:10.3390/jpm11050381)
Supplement: Supplementary file 1 [file jpm-11-00381-s001.zip › jpm-1198739-supplementary.pdf]

Supplementary Materials

Table S1. PA levels (MET-min/week) and sitting/lying time (h:min/day) by sociodemographic characteristics, region of residence, number of family members, dwelling floor space, private outdoor spaces, residential density, self-reported PA strategies, anthropometric parameters, quality of life, and health perception and use of psychotropic drugs of the study population during quarantine. Data are expressed as the means ± SD and percent of respondents engaged in PA or in sitting/lying.

	Vigorous intensity PA	Moderate intensity PA	Walking	Total PA	Sitting/lying
Age					
**21–40 years**	224.44 ± 531.97<br>22.2%	873.89 ± 996.68<br>91.7%	227.24 ± 614.32<br>44.4%	1325.57 ± 1460.79<br>94.4%	7:51 ± 3:00
41–60 years	124.98 ± 487.14<br>11.9%	849.57 ± 896.38<br>89.9%	330.40 ± 564.86<br>61.0%	1304.95 ± 1262.09<br>94.0%	7:20 ± 3:09
Over 60	74.73 ± 390.37<br>7.7%	1016.59 ± 1052.06<br>88.5%	357.09 ± 660.65<br>62.6%	1448.41 ± 1403.37<br>93.4%	6:25 ± 3:01*
Pearson's corr. coeff.	*--0.084**	*0.067**	*0.045*	*0.038*	*-0.150**
Marital status					
**Never married**	150.42 ± 593.37<br>14.8%	634.23 ± 767.22<br>89.4%	345.82 ± 622.55<br>65.5%	1130.47 ± 1253.82<br>95.1%	7:30 ± 3:20
Widowed	137.14 ± 551.59<br>9.5%	1001.90 ± 1092.45<br>81.0%	315.81 ± 709.93<br>52.4%	1454.86 ± 1554.58<br>85.7%	6:24 ± 2:59
Separated/divorced	118.26 ± 462.61<br>12.0%	905.65 ± 825.16<br>94.6%	314.03 ± 449.17<br>65.2%	1337.95 ± 1011.06<br>98.9%	6:51 ± 2:53
Married	107.98 ± 427.60<br>10.5%	949.66 ± 985.83*<br>89.0%	331.23 ± 599.48<br>58.7%	1388.87 ± 1351.27<br>92.8%	7:09 ± 3:08
Spearman's Rho	*-0.046*	*0.105**	*-0.032*	*1.000*	*-0.015*
Levels of education					
**≤13 y**	102.96 ± 519.69<br>7.7%	951.22 ± 966.23<br>90.6%	295.95 ± 541.72<br>59.4%	1350.13 ± 1302.91<br>93.9%	6:53 ± 3:06
>13 y	132.54 ± 411.29<br>15.2%	825.35 ± 911.51<br>88.4%	367.20 ± 634.27<br>61.7%	1325.10 ± 1309.68<br>93.6%	7:25 ± 3:09*
Working activity					
**Retired or laid off**	71.63 ± 345.63<br>8.4%	935.16 ± 970.80<br>87.0%	402.52 ± 720.61<br>60.9%	1409.31 ± 1376.70<br>92.6%	6:09 ± 2:53
Remote working	143.98 ± 454.40<br>14.5%	788.12 ± 836.46<br>91.2%	293.52 ± 512.56<br>60.5%	1225.62 ± 1179.74<br>94.9%	8:02 ± 3:04*
Normal working	47.78 ± 163.57<br>9.7%	668.89 ± 753.60<br>86.1%	355.33 ± 637.08<br>56.9%	1071.99 ± 1158.19<br>91.7%	7:00 ± 3:10
Other	157.75 ± 705.54<br>9.2%	1178.17 ± 1140.82*<br>90.8%	305.69 ± 518.74<br>62.0%	1641.61 ± 1497.78<br>93.7%	6:35 ± 3:06
Spearman's Rho	*0.014*	*0.047*	*-0.012*	*0.028*	*0.070*

Region of residence					
**Northern Italy**	121.25 ± 500.51<br>11.1%	853.68 ± 900.50<br>89.2%	327.28 ± 583.58<br>60.1%	1302.21 ± 1284.89<br>93.4%	7:10 ± 3:09
Central Italy	144.06 ± 423.33<br>15.6%	910.78 ± 968.64<br>90.6%	300.70 ± 483.67<br>65.6%	1355.54 ± 1304.86<br>96.1%	7:02 ± 3:07
Southern Italy	47.27 ± 232.94<br>6.5%	1112.21 ± 1149.70*<br>89.6%	413.64 ± 774.91<br>55.8%	1573.12 ± 1445.60<br>92.2%	7:17 ± 3:06
Spearman's Rho	*0.004*	*0.059*	*0.006*	*0.056*	*0.000*
Number of family members					
**1 member**	106.61 ± 496.34<br>9.8%	742.50 ± 827.53<br>91.1%	1067.92 ± 867.98<br>66.1%	2363.72 ± 1811.20<br>95.1%	7:18 ± 3:09
2 members	132.42 ± 517.04<br>12,1%	929.11 ± 976.50<br>87.9%	918.29 ± 860.74<br>54.4%	2196.68 ± 1683.66<br>92.7%	7:11 ± 3:12
3 or more members	113.92 ± 404.33<br>12.0%	961,81 ± 979.36*<br>89.6%	867.77 ± 798.40<br>61.5%	2116.50 ± 1615.83<br>93.5%	7:01 ± 3:05
Spearman's Rho	*0.025*	*0.090**	*-0.013*	*0.054*	*-0.036*
Dwelling floor space					
**≤ 50 m^2**	180.44 ± 898.43<br>8.9%	529.33 ± 511.28<br>91.1%	1157.64 ± 947.99<br>62.2%	2521.64 ± 2151.55<br>93.3%	8:00 ± 3:24
50 ≤ 90 m^2	109.28 ± 434.16<br>10.5%	813.14 ± 859.79<br>88.9%	952.44 ± 842.22<br>61.4%	2235.77 ± 1724.21<br>92.5%	7:21 ± 3:06
> 90 m^2	117.12 ± 426.95<br>12.3%	979.77 ± 1015.50*<br>89.8%	910.58 ± 828.24<br>59.8%	2164.25 ± 1621.54<br>94.7%	6:56 ± 3:07*
Spearman's Rho	*0.033*	*0.113**	*0.003*	*0.096**	*-0.087**
Private outdoor spaces					
**None**	174.17 ± 590.22<br>12.5%	647.92 ± 861.53<br>89.6%	772.13 ± 783.31<br>64.6%	1706.71 ± 1315.88<br>93.8%	7:55 ± 3:29
Balcony	109.36 ± 467.57<br>10,6%	828.78 ± 872.33<br>88.7%	1006.13 ± 887.24<br>59.2%	2236.60 ± 1774.89<br>93.1%	7:20 ± 3:04
Garden	121.42 ± 448.27<br>12.4%	1025.04 ± 1039.33*<br>90.8%	866.17 ± 765.79<br>62.1%	2261.07 ± 1613.65<br>94.7%	6:44 ± 3:10*
Spearman's Rho	*0.018*	*0.127**	*0.064*	*0.132**	*-0.117**
Residential density					
**Cities**	103.91 ± 413.67<br>9.7%	811.47 ± 914.83<br>90,1%	907.52 ± 794.06<br>60.9%	2083.23 ± 1616.63<br>94.1%	7:23 ± 3:13
Suburbs	124.60 ± 456.08<br>12.3%	931.49 ± 921.69<br>91.1%	1047.10 ± 916.37<br>58.7%	2429.91 ± 1821.68<br>95.3%	7:05 ± 2:57
Countryside	138.03 ± 585.28<br>13.9%	996.30 ± 1010.78<br>86.1%	870.03 ± 827.34*<br>62.4%	2197.54 ± 1666.64*<br>90.8%	6:44 ± 3:12*
Spearman's Rho	*0.052*	*0.103**	*0.044*	*0.107**	*-0.080**
PA strategies					
**None**	117.07 ± 477.43<br>12.1%	894.38 ± 937.91<br>90.5%	337.87 ± 614.19<br>61.2%	1349.31 ± 1293.56<br>94.3%	7:10 ± 3:05
Some	118.84 ± 453.36<br>10.1%	877.83 ± 947.91<br>87.7%	319.68 ± 544.86<br>59.4%	1316.35 ± 1329.19<br>92.8%	7:07 ± 3:14

Waist circumference					
Normal	156.70 ± 527.23<br>14.8%	837.95 ± 893.14<br>90.6%	369.84 ± 632.04<br>62.6%	1364.50 ± 1323.23<br>95.1%	7:05 ± 3:07
Abdominal obesity	64.94 ± 369.81<br>6.9%	956.93 ± 999.14*<br>88.1%	279.50 ± 525.19<br>57.9%	1301.37 ± 1282.24<br>91.9%	7:14 ± 3:11
Pearson's corr. coeff.	*-0.001*	*-0.066*	*0.380*	*-0.045*	*0.004*
BMI					
Underweight	65.66 ± 219.24<br>11.3%	878.11 ± 816.27<br>94.3%	409.14 ± 718.55<br>58.5%	1352.92 ± 1234.69<br>96.2%	6:41 ± 3:13
**Normal weight**	149.02 ± 534.56<br>13.6%	854.89 ± 920.43<br>90.3%	354.61 ± 600.44<br>64.0%	1358.51 ± 1334.14<br>94.5%	7:09 ± 3:09
Overweight and obese	48.80 ± 289.73*<br>5.5%	980.10 ± 1020.15<br>86.0%	249.69 ± 516.25<br>52.0%	1278.59 ± 1250.44<br>91.0%	7:18 ± 3:05
Pearson's corr. coeff.	*-0.068*	*0.045*	*-0.092**	*-0.033*	*0.028*
Lymphedema					
**No**	116.26 ± 462.23<br>11.7%	894.01 ± 947.50<br>89.8%	338.00 ± 599.08<br>61.2%	1348.28 ± 1299.62<br>93.8%	7:11 ± 3:11
Yes	129.30 ± 521.49<br>9.3%	844.19 ± 889.60<br>87.2%	278.40 ± 514.21<br>55.8%	1251.88 ± 1356.91<br>93.0%	6:50 ± 2:47
Quality of life					
Very poor	0.00 ± 0.00<br>7.4%	402.00 ± 496.52<br>74.1%	153.45 ± 328.06*<br>51.9%	555.45 ± 524.09<br>77.8%	9:32 ± 3:15*
Poor	116.36 ± 505.44<br>7.5%	796.82 ± 1018.25<br>86.3%	217.18 ± 367.87*<br>50.7%	1130.36 ± 1184.55<br>91.1%	7:21 ± 3:08*
Neither poor nor good	71.60 ± 343.87<br>9.5%	935.29 ± 925.60<br>89.9%	335.19 ± 632.01*<br>58.5%	1342.08 ± 1252.79<br>94.4%	6:54 ± 3:04
Good	134.25 ± 521.40<br>14.9%	870.97 ± 935.63<br>92.4%	341.13 ± 584.81<br>66.5%	1346.36 ± 1352.76<br>95.6%	7:10 ± 3:06
**Very good**	194.71 ± 504.49<br>22.2%	964.41 ± 1016.31<br>88.9%	358.43 ± 619.90<br>85.2%	1517.55 ± 1314.58<br>96.3%	7:30 ± 3:29
Spearman's Rho	*0.111**	*0.067*	*0.191**	*0.146**	*-0.093**
Health perception					
Very poor	0.00 ± 0.00*<br>0.00%	332.50 ± 414.72<br>50.0%	173.88 ± 307.02<br>50.0%	506.38 ± 702.54<br>50.0%	10:45 ± 2:36*
Poor	120.00 ± 692.59<br>6.2%	842.65 ± 1033.74<br>81.4%	246.49 ± 511.67<br>46.9%	1209.14 ± 1461.38<br>88.5%	7:19 ± 2:59
Neither poor nor good	66.67 ± 285.89<br>7.7%	928.13 ± 976.38<br>88.6%	311.41 ± 587.97<br>60.8%	1306.21 ± 1247.26<br>93.4%	7:04 ± 3:08
Good	148.97 ± 494.59<br>14.7%	898.01 ± 917.71<br>93.5%	361.81 ± 615.41<br>63.0%	1408.79 ± 1324.87<br>96.2%	7:07 ± 3:09
**Very good**	203.48 ± 489.52<br>23.9%	792.61 ± 672.79<br>91.3%	461.30 ± 611.49<br>76.1%	1457.39 ± 1123.08<br>97.8%	6:47 ± 3:16
Spearman's Rho	*0.146**	*0.068*	*0.130**	*0.121**	*-0.044*
Psychotropic drugs					
**No**	127.35 ± 488.96	876.48 ± 916.74	335.90 ± 600.02	1339.73 ± 1289.99	7:09 ± 3:08

	12.1%	89.9%	60.2%	93.9%	
Yes	68.44 ± 345.45<br>7.8%	950.00 ± 1057.29<br>87.5%	308.70 ± 539.80<br>62.5%	1327.14 ± 1387.23<br>93.0%	7:11 ± 3:11

* Student *t*-test or ANOVA (Dunnet *post-hoc*); p <0.05. Control groups are in bold.

Notes: PA = Physical Activity.
